# Rosmarinic Acid Methyl Ester Regulates Ovarian Cancer Cell Migration and Reverses Cisplatin Resistance by Inhibiting the Expression of Forkhead Box M1

**DOI:** 10.3390/ph13100302

**Published:** 2020-10-12

**Authors:** Soo Hyun Lim, Ki Hong Nam, Kyungtae Kim, Sang Ah Yi, Jaecheol Lee, Jeung-Whan Han

**Affiliations:** 1 School of Pharmacy, Sungkyunkwan University, Suwon 16419, Korea; barbie01096@naver.com (S.H.L.); nam6422@hanmail.net (K.H.N.); fate514@naver.com (K.K.); angelna1023@hanmail.net (S.A.Y.); jaecheol@skku.edu (J.L.); 2 Biomedical Institute for Convergence at SKKU (BICS), Sungkyunkwan University, Suwon 16419, Korea; 3 Imnewrun Biosciences Inc., Suwon 16419, Korea

**Keywords:** rosmarinic acid methyl ester, ovarian cancer, FOXM1, migration, invasion, cisplatin resistance

## Abstract

Rosmarinic acid methyl ester (RAME), a derivative of rosmarinic acid (RA), is reported to have several therapeutic effects, including anti-tumor effects against cervical cancer. However, its anti-tumor effects in ovarian cancer is unclear. In this study, we studied the molecular pathways associated with the anti-tumor effects of RAME in ovarian cancer. To identify the effects of RAME in ovarian cancer, RNA sequencing was performed in RAME-treated ovarian cancer cells; we found that RAME treatment downregulated the genes closely involved with the target genes of the transcription factor Forkhead box M1 (FOXM1). It was reported that FOXM1 is overexpressed in a variety of cancer cells and is associated with cell proliferation and tumorigenesis. Therefore, we hypothesized that FOXM1 is a key target of RAME; this could result in its anti-tumor effects. Treatment of ovarian cancer cells with RAME-inhibited cell migration and invasion, as shown by wound healing and transwell migration assays. To examine whether RAME represses the action of FOXM1, we performed quantitative RT-PCR and ChIP-qPCR. Treatment of ovarian cancer cells with RAME decreased the mRNA expression of FOXM1 target genes and the binding of FOXM1 to its target genes. Moreover, FOXM1 expression was increased in cisplatin-resistant ovarian cancer cells, and combination treatment with RAME and cisplatin sensitized the cisplatin-resistant ovarian cancer cells, which was likely due to FOXM1 inhibition. Our research suggests that RAME is a promising option in treating ovarian cancer patients, as it revealed a novel molecular pathway underlying its anti-tumor effects.

## 1. Introduction

Ovarian cancer, the most lethal gynecological cancer, is the second most common malignancy after breast cancer in women over the age of 40 [1]. About 80% of all ovarian cancers are of the high-grade serous type, arising from the serous epithelial layer in the abdominopelvic cavity [2]. Since most ovarian cancers are diagnosed at advanced stages, they have a poor prognosis, and a low overall survival rate [3]. Moreover, the survival rate of patients with ovarian cancer has barely changed since platinum-based treatment was introduced more than 30 years ago [4]. Therefore, there is an important need for new strategies to treat ovarian cancers and a better understanding of the molecular events leading to the resistance of treatment.

Forkhead box M1 (FOXM1), a member of the Forkhead box transcription factor family, is an oncogenic transcription factor, and its overexpression is associated with poor prognosis in several types of human cancers, such as pancreatic cancer, breast cancer, and lung cancer [5,6,7]. FOXM1 is involved in cell cycle progression through regulation of gene expression in the G1/S and G2/M phases and by inducing the proper execution of the mitotic program [8,9,10]. Moreover, FOXM1 plays an important role in the early stage of metastasis, by stimulating the expression of genes associated with the invasion and migration of cancer cells [11,12,13]. FOXM1 also increases the population, proliferation, and motility of cancer stem cells, which make cancers tolerant to drugs like cisplatin [14,15]. Hence, targeting FOXM1 could be effective for treating several cancers and sensitizing drug-resistant cancer cells.

Rosmarinic acid methyl ester (RAME), a derivative of rosmarinic acid (RA), has several biological effects, such as anti-microbial, anti-inflammatory, and anti-allergic effects [16,17,18]. RAME also exhibits anti-oxidant and anti-melanogenesis in vivo [19]. Recently, the anti-cancer effect of RAME via the inhibition of mTOR-S6K1 signaling in cervical cancer was reported [20]. It was also reported that RAME induced the apoptosis of cervical cancer cells and enhanced the anti-tumor effect of cisplatin in cervical cancer. However, the regulation of gene expression by RAME treatment and the mechanisms through which gene expression is regulated, are unclear.

Here, we performed RNA-sequencing in RAME-treated ovarian cancer cells. Differentially Expressed Gene (DEG) analysis and Gene Ontology (GO) analysis suggest that the expression of mitosis-associated genes known to be regulated by FOXM1 were downregulated. Treatment of ovarian cancer cells with RAME effectively inhibited the binding of FOXM1 to its target gene promoters by decreasing the FOXM1 expression. We also observed that RAME repressed the migration and invasion of ovarian cancer cells. Moreover, co-treatment with RAME and cisplatin sensitized a cisplatin-resistant ovarian cancer cell line and induced the expression of apoptosis-associated genes. Collectively, our studies suggested that RAME, a derivative of rosmarinic acid, has the potential for use as a therapeutic substance for patients with ovarian cancer, especially for those with cisplatin-resistant tumors.

## 2. Results

### 2.1. Transcriptome Analysis of Ovarian Cancer Cells Treated with RAME Shows that FOXM1 Target Genes are Downregulated

**Figure 1:**
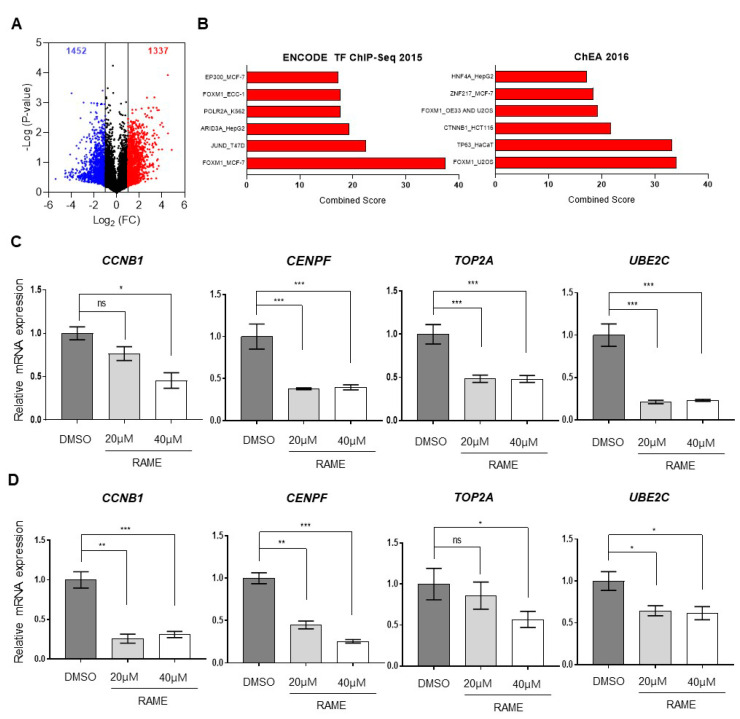
Transcriptome analysis of SKOV-3 cells treated with RAME shows that FOXM1 target genes are downregulated. (**A**) RNA-sequencing data with RAME (40 µM)-treated SKOV-3 ovarian cancer cells. (**B**) Gene Ontology (GO) analysis of RNA-sequencing data using the Enrichr tool. (**C**) The mRNA expression levels of FOXM1 target genes in SKOV-3 cells treated with RAME (20 µM and 40 µM) for 24 h. (**D**) The mRNA expression levels of FOXM1 target genes in TOV-21G cells treated with RAME (20 µM and 40 µM) for 24 h. Error bars correspond to mean ± SEM (*n* = 3). * *p* < 0.05, ** *p* < 0.01, *** *p* < 0.001; unpaired *t*-test.

Previously, it was reported that RAME induced the autophagy and apoptosis of cervical cancer cells [20]. To further elucidate the molecular pathways demonstrating its anti-tumor effects against ovarian cancer, we performed the RNA-sequencing of RAME-treated SKOV-3 ovarian cancer cells. Since 40 µM of RAME treatment showed apoptotic effects in cervical cancer cells [20], we treated another ovarian cancer cell line, SKOV-3, with 40 µM of RAME. As shown by the RNA-sequencing data, 2789 differentially expressed genes (DEGs) were identified, including 1337 upregulated genes and 1452 downregulated genes (Figure 1A). We selected 242 downregulated genes (|log_2_ (Fold change)| > 1, *p*-value < 0.05), and gene ontology (GO) analysis of the RNA-sequencing data using the Enrichr tool revealed that the RAME treatment downregulated the target genes of the transcription factor Forkhead box M1 (FOXM1) (Figure 1B, Supplementary Table S1). In ovarian cancer, the gene expression level of FOXM1 was higher than normal tissue (Figure S1). FOXM1 promotes cell proliferation and migration by regulating the expression of its target genes [21,22,23,24,25,26,27]. These target genes included CCNB1, CENPF, TOP2A, and UBE2C, and were downregulated, as per our RNA-sequencing data. Therefore, we confirmed the mRNA expression levels of FOXM1-targeted genes following RAME treatment for 24 h, using RT-qPCR. SKOV-3 cells were treated with various doses of RAME, and the mRNA expression levels of CCNB1, CENPF, TOP2A, and UBE2C decreased significantly in a dose-dependent manner (Figure 1C). In addition, the mRNA expression levels of FOXM1 target genes were decreased in RAME-treated TOV-21G cells (Figure 1D). Altogether, these data indicated that RAME treatment suppressed the expression of FOXM1 target genes that were associated with enhancing cell migration in ovarian cancer cells.

### 2.2. RAME Inhibits Expression of FOXM1 and its Interaction with Target Genes

**Figure 2:**
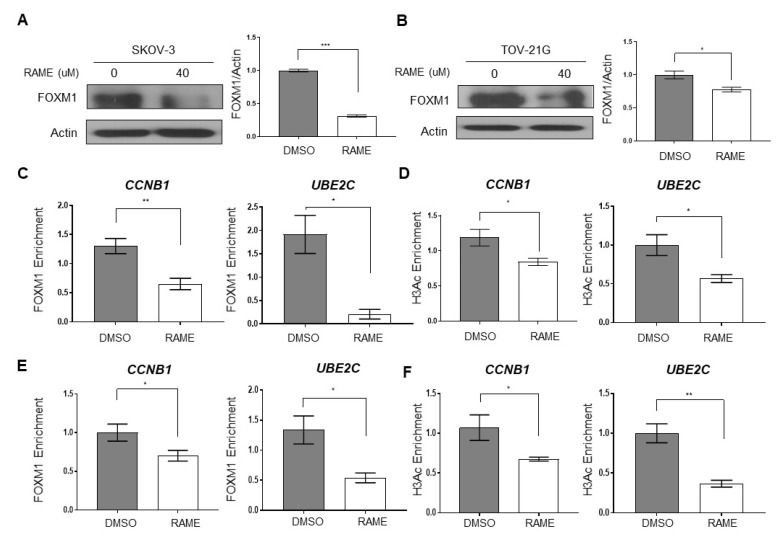
RAME decreases FOXM1 levels and the expression of its target genes by inhibiting the enrichment of FOXM1 on promoters. (**A**) Immunoblot analysis of FOXM1 (left) and its quantitative graph (right) in SKOV-3 cells. (**B**) Immunoblot analysis of FOXM1 (left) and its quantitative graph (right) in TOV-21G cells. (**C**,**D**) Chromatin Immunoprecipitation (ChIP)-qPCR analysis with FOXM1 (**C**) and H3Ac (**D**) antibody on CCNB1 and UBE2C promoters in RAME-treated SKOV-3 cells treated with 40 µM of RAME for 24 h. (**E**,**F**) Chromatin Immunoprecipitation (ChIP)-qPCR analysis with FOXM1 (**E**) and H3Ac (**F**) antibody on CCNB1 and UBE2C promoters in RAME-treated TOV-21G cells treated with 40 µM of RAME for 24 h. Error bars represent the mean ± SEM (*n* = 3). * *p* < 0.05, ** *p* < 0.01, *** *p* < 0.001; unpaired *t*-test.

To figure out whether RAME inhibits the mRNA expression of the FOXM1 target genes by regulating FOXM1 expression, we investigated FOXM1 protein levels in ovarian cancer cell lines treated with RAME. Immunoblot analyses showed that treatment with 40 µM RAME for 24 h decreased the protein levels of FOXM1 in SKOV-3 and TOV-21G cells (Figure 2A,B). We next assessed the direct binding of FOXM1 to the promoters of the downregulated genes through chromatin immunoprecipitation (ChIP)-qPCR using FOXM1 antibodies. The ChIP-qPCR analysis indicated that RAME treatment (40 μM) decreased the enrichment of FOXM1 at the promoters of CCNB1 and UBE2C (Figure 2C,E). Furthermore, ChIP-qPCR using H3 acetylation (H3Ac) antibodies was performed to unravel the epigenetic regulatory mechanisms. H3 acetylation at the promoters of CCNB1 and UBE2C was reduced following the RAME treatment, causing a downregulation in gene expression (Figure 2D,F). These results indicated that RAME reduced FOXM1 target gene expression via the regulation of FOXM1 expression and the occupancy of FOXM1 at the promoters of FOXM1 target genes, as well as by inhibition of H3 acetylation.

### 2.3. RAME Inhibits Cell Migration and Invasion in Ovarian Cancer Cell Line

**Figure 3:**
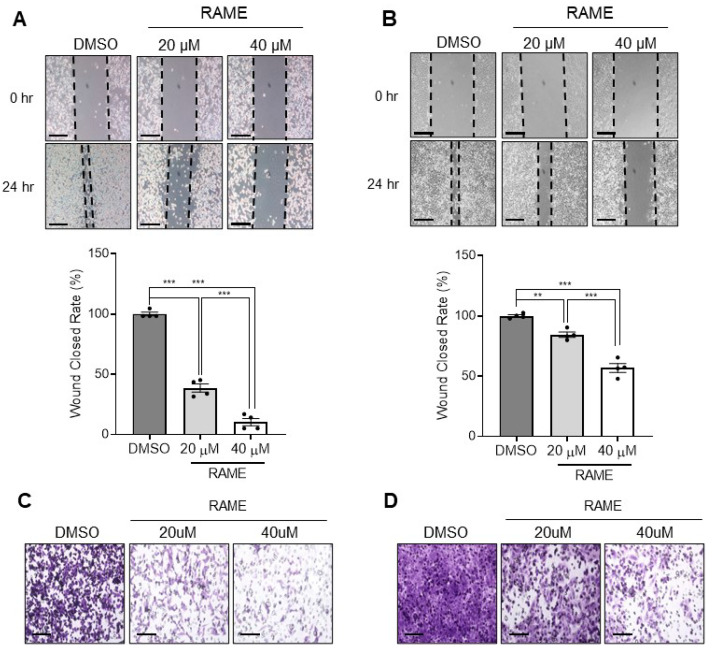
RAME inhibits cell migration and invasion in an ovarian cancer cell line. (**A**) Gaps of SKOV-3 after 24 h of RAME treatment at concentrations of 0, 20, and 40 µM (upper) and its quantitative wound close rate (lower). Scale bar = 200 μm. (**B**) Gaps of TOV-21G after 24 h of RAME treatment at concentrations of 0, 20, and 40 µM (upper) and its quantitative wound close rate (lower). Scale bar = 200 μm. (**C**) Invaded cells after RAME treatment for 24 h at concentrations of 0, 20, and 40 µM in SKOV-3 cells. Scale bar = 100 μm. (**D**) Invaded cells after RAME treatment for 24 h at concentrations of 0, 20, and 40 µM in TOV-21G cells. Scale bar = 100 μm. Error bars represent the mean ± SEM (*n* = 3). ** *p* < 0.01, *** *p* < 0.001; unpaired *t*-test.

FOXM1 target genes are known to enhance cell migration and invasion [28,29]. Since RAME inhibits the expression of FOXM1 and its target genes, we hypothesized that RAME would also decrease the migration and invasion abilities of ovarian cancer cells. To assess the impact of RAME on the migration of ovarian cancer cell lines, we performed a wound healing assay. It was found that RAME reduced the migration ability of TOV-21G ovarian cancer cells in a dose-dependent manner (Figure 3A). Moreover, the migration ability of the SKOV-3 cells was also suppressed dose-dependently, following RAME treatment for 24 h (Figure 3B). Likewise, the transwell invasion assay revealed that RAME treatment inhibited the invasion of TOV-21G cells in a dose-dependent manner (Figure 3C). Furthermore, the invasion ability of SKOV-3 cells was also reduced significantly, as the concentration of RAME increased (Figure 3D). The inhibition of migration and invasion by RAME treatment were not due to apoptosis, in that, the RAME treatment did not increase the apoptosis marker gene expression in the SKOV-3 (Figure S2A) and TOV-21G cells (Figure S2B). These results suggest that the motility of ovarian cancer cells is repressed by RAME treatment.

### 2.4. RAME Regulates Target Gene Expression via FOXM1

**Figure 4:**
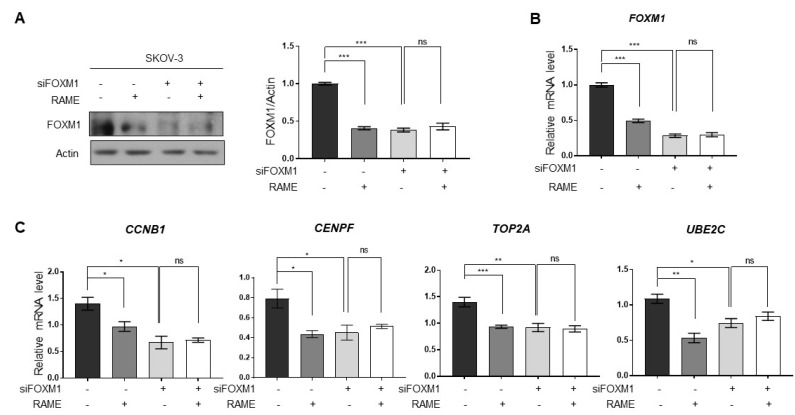
RAME regulates target gene expression via FOXM1. (**A**) Immunoblot analysis of SKOV-3 cells and FOXM1-knockdown-SKOV-3 cells following RAME treatment, as indicated. (**B**) The mRNA levels of FOXM1 in SKOV-3 cells and FOXM1-knockdown-SKOV-3 cells following RAME treatment, as indicated. (**C**) The mRNA levels of FOXM1 target genes in SKOV-3 and FOXM1-knockdown-SKOV-3 cells following RAME treatment, as indicated. Error bars represent the mean ± SEM (*n* = 3). * *p* < 0.05, ** *p* < 0.01, *** *p* < 0.001; unpaired *t*-test.

RAME inhibited FOXM1 target genes by downregulating FOXM1 and its binding with its target genes (Figure 1C,D and Figure 2). To ascertain whether RAME regulates the expression of FOXM1 target genes via FOXM1 inhibition, not other molecular pathways, SKOV-3 ovarian cancer cells were transfected with siFOXM1 for knocking down FOXM1. As shown by immunoblot analysis, FOXM1 levels were reduced in RAME-treated (40 µM) SKOV-3 cells. On the other hand, RAME did not reduce the FOXM1 levels in the FOXM1-knockdown cells (Figure 4A). Likewise, the mRNA levels of FOXM1 were also suppressed in SKOV-3 cells after RAME (40 µM) treatment, but not in RAME-treated FOXM1-knockdown cells (Figure 4B). In addition, RAME treatment suppressed the expression of FOXM1 target genes. Conversely, there was no change in the expression of FOXM1 target genes in FOXM1-knockdown cells, following RAME treatment (Figure 4C). Collectively, these results revealed that FOXM1 target genes are regulated via FOXM1 and not the other pathways.

### 2.5. RAME Accelerates Anticancer Drug Effects in Cisplatin Resistant Ovarian Cancer Cell Line

**Figure 5:**
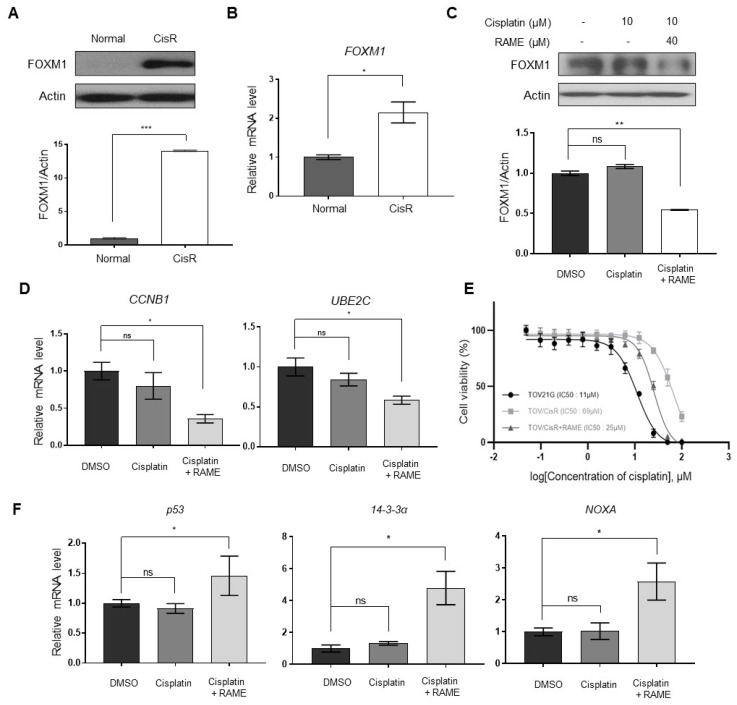
RAME sensitizes cisplatin-resistant ovarian cancer cells. (**A**) Immunoblot analysis of TOV-21G and cisplatin-resistant TOV-21G cells and its quantitative graph. (**B**) The mRNA levels of the Foxm1 gene in TOV-21G and cisplatin-resistant TOV-21G cells. (**C**) Immunoblot analysis of cisplatin-resistant TOV-21G cells and its quantitative graph. (**D**) The mRNA levels of Foxm1-binding genes in cisplatin-resistant TOV-21G cells. (**E**) Cell viability assay curves for cisplatin and the half maximal inhibitory concentration (IC50) values of TOV-21G and cisplatin-resistant TOV-21G cells. (**F**) The mRNA levels of apoptotic genes in cisplatin-resistant TOV-21G cells. Error bars represent the mean ± SEM (*n* = 3). * *p* < 0.05, ** *p* < 0.01, *** *p* < 0.001; unpaired *t*-test.

It was reported that cisplatin-resistant cancer cell lines showed elevated FOXM1 levels [30]. To confirm this, we established cisplatin resistant ovarian cancer cell line using TOV-21G cells, which have low FOXM1 expression than SKOV-3 cell (Figure S3) and performed an immunoblotting assay in TOV-21G and cisplatin-resistant TOV-21G cells. In the cisplatin-resistant TOV-21G cell line, FOXM1 levels were considerably elevated, compared to the TOV-21G cell line (Figure 5A,B). Within this cisplatin-resistant TOV-21G cell line, we checked the differences in FOXM1 levels after treatment with cisplatin and RAME. Although the cisplatin-only treated group did not show any changes in the FOXM1 levels, the cells treated with cisplatin and RAME showed reduced FOXM1 levels (Figure 5C). We also confirmed that the downregulation of FOXM1 by RAME reduced the mRNA expression levels of the FOXM1-binding genes CCNB1 and UBE2C (Figure 5D). The cells treated with both cisplatin and RAME showed reduced viability, compared to those treated with cisplatin only (data not shown); hence, we performed cell viability assays of the TOV-21G cell line under several conditions. The cisplatin-resistant TOV-21G cell line showed a higher IC50 value than the normal TOV-21G cell line, but this IC50 value was reduced in the presence of RAME (Figure 5E). Moreover, in the cisplatin-resistant TOV-21G cell line, genes related to cell apoptosis showed increased mRNA expression levels, only following treatment with both cisplatin and RAME (Figure 5F). In conclusion, RAME induced accelerated anti-cancer effects by regulating FOXM1 expression levels, and it could be considered to be a potential anticancer therapeutic.

## 3. Discussion

Several studies revealed that FOXM1 is overexpressed in various cancer cells, such as ovarian, breast, lung, and cervical cancer cells [12,30,31,32]. Upregulation of FOXM1 enhances cell proliferation and inhibits apoptosis [14,33,34]. Moreover, upregulated FOXM1 is related to poor prognosis in several cancers [12,35]. Therefore, FOXM1 is a promising therapeutic target for inducing anti-cancer effects in various cancer cells [36,37]. In this study, we hypothesized that RAME could exert anti-cancer effects since it downregulated the expression of FOXM1 target genes, according to our RNA sequencing data. Based on this hypothesis, we investigated the expression of FOXM1 target genes and the recruitment of FOXM1 onto the promoters of its target genes. We identified that RAME suppressed the expression of FOXM1 target genes by inhibiting the enrichment of FOXM1 on their promoters, as shown in Figure 1C,D and Figure 2. Moreover, by suppressing the expression of the FOXM1 and its target genes, the migration and invasion abilities of ovarian cancer cells were suppressed (Figure 3).

Ovarian cancer is commonly diagnosed in the advanced stages; however, ovarian cancer is more curable at the early stages than at the later stages [38]. This is due to the lack of warning signs or obvious symptoms in early-stage ovarian cancer; hence, ovarian cancer is usually called “the silent killer” [38,39,40]. Unfortunately, the diagnosis of ovarian cancer at advanced stages leads to the acquisition of resistance to conventional chemotherapy and poor prognosis [41,42]. For these reasons, there is an increasing need for an effective treatment against chemotherapy-resistant ovarian cancer.

Cisplatin is one of the conventional agents used to treat ovarian cancer. Its anti-cancer effect is a result of DNA damage-induced apoptosis [43]. Ovarian cancer often develops cisplatin resistance after cisplatin chemotherapy [44]. Furthermore, recurrence of ovarian cancer is up to 75% and the recurrent cancer can result in the acquisition of cisplatin resistance, as well [43]. Thus, cisplatin resistance is a limitation of cisplatin chemotherapy, since there are several molecular mechanisms leading to cisplatin resistance [45,46,47]. Among the mechanical reasons, previous studies showed that upregulation of FOXM1 induced cisplatin resistance in several cancer cells [48,49,50]. From these previous studies, we hypothesized that RAME could overcome cisplatin resistance in ovarian cancer cells. Our data revealed that co-treatment with RAME and cisplatin was effective against cisplatin-resistant cancer cells by inhibiting the expression of FOXM1 and its target genes, ultimately inducing apoptosis in these cells (Figure 5).

## 4. Materials and Methods

### 4.1. Antibodies and Reagents

Anti-FOXM1 (GeneTex, Irvine, CA, USA; GTX102170) and anti-β-Actin (Millipore, Temecula, CA, USA; mab1501) antibodies were utilized for the immunoblotting assay in this study. In addition, for the Chromatin Immunoprecipitation assay, anti-FOXM1 (GeneTex, Irvine, CA, USA; GTX102170) and anti-H3Ac (Millipore, Temecula, CA, USA; 06-599) antibodies were used. Rosmarinic acid methyl ester (RAME) was purchased from Chemfaces (Wuhan, China; No. CFN97567). RAME was extracted from the herbs of *Rosmarinus officinalis* L. and its purity was ≥98%.

### 4.2. Cell Culture and Establishment of Cisplatin-Resistant TOV-21G (TOV/CisR) Cells

SKOV-3, a human ovarian cancer cell line, was maintained in McCoy’s 5A medium supplemented with 10% fetal bovine serum (FBS), 1% penicillin/streptomycin (P/S) (100×). Additionally, another ovarian cancer cell line, TOV-21G, was grown in Dulbecco’s Modified Eagle’s Medium (DMEM) supplemented with 10% FBS and 1% P/S (100×). These cell types were obtained from the American Type Culture Collection (ATCC) and maintained in a humidified atmosphere (37 °C, 5% CO_2_). Cisplatin-resistant TOV-21G (TOV/CisR) cells were established by culturing the cells with gradually increasing concentrations of cisplatin [51].

### 4.3. RNA Isolation, Library Preparation, and RNA-Sequencing

The total RNA of SKOV-3 cells was isolated using the Trizol reagent (Invitrogen, Carlsbad, CA, USA). For the gene expression profiling, RNA-sequencing was performed at Ebiogen Inc. (Seoul, Korea). RNA quality was assessed with an Agilent 2100 Bioanalyzer using the RNA 6000 Nano Chip (Agilent Technologies, Amstelveen, The Netherlands), and RNA quantification was performed using an ND-2000 Spectrophotometer (Thermo Fisher Scientific, Waltham, MA, USA). For the RNAs of DMSO- and RAME-treated SKOV-3 cells, a library was constructed using the QuantSeq 3′ mRNA-Seq Library Prep Kit (Lexogen Inc., Vienna, Austria), according to the manufacturer’s instructions. In brief, 500 ng of total RNA was prepared and an oligo-dT primer containing an Illumina-compatible sequence at its 5′ end was hybridized to the RNA; reverse transcription was then performed. After degradation of the RNA template, second strand synthesis was initiated by a random primer containing an Illumina-compatible linker sequence at its 5′ end. The double-stranded library was purified using magnetic beads to remove all reaction components. The library was amplified to add the complete adapter sequences required for cluster generation. The finished library was purified from the PCR components. High-throughput sequencing was performed as single-end 75 sequencing using NextSeq 500 (Illumina Inc., San Diego, CA, USA). QuantSeq 3′ mRNA-Seq reads were aligned using Bowtie2 [52]. Bowtie2 indices were either generated from the genome assembly sequence or the representative transcript sequences for alignment to the genome and transcriptome, respectively. The alignment file was used for assembling transcripts, estimating their abundance, and detecting the differential expression of genes. Analysis of the relationship between differentially expressed genes was performed with the Excel-based Differentially Expressed Gene Analysis (ExDEGA v.2.0.0) software by eBiogen (ebiogen.com). Differentially expressed genes with fold changes >2 and *p*-values < 0.05 were further identified by GO analysis using the Enrichr tool [53,54].

### 4.4. RNA Extraction and Quantative Real-Time PCR (qPCR)

Total RNA was isolated from the cells using an Easy-Blue reagent (iNtRON Biotechnology, Seongnam, Korea). Next, 1 μg of RNA was reverse-transcribed using a Maxim RT-PreMix Kit (iNtRON Biotechnology, Seongnam, Korea). Quantitative real-time PCR (qPCR) was performed using a KAPA^TM^ SYBR^®^ FAST qPCR Master Mix (Kapa Biosystems, Wilmington, MA, USA) and CFX96 TouchTM real-time PCR detector (Bio-Rad, Hercules, CA, USA). The relative mRNA expression levels of the target genes were normalized to the mRNA levels of GAPDH for each reaction. The primer sequences used for RT-qPCR were as follows: *FOXM1* forward, 5′-AACCGCTACTTGACATTG G-3′; *FOXM1* reverse, 5′-GCAGTGGCTTCATCTTCC-3′; *CCNB1* forward, 5′-CCAGTGCCAGTGTCTGAGC-3′; *CCNB1* reverse, 5′-TGGAGAGGCAGTATCAACCA-3′; *CENPF* forward, 5′-CAAGAATATGCACAACGTCCTGC-3′; *CENPF* reverse, 5′ GAACGCCTGTTCAGCTCTG-3′; *TOP2A* forward, 5′-CTGCGGACAACAAACAAAGG-3′; *TOP2A* reverse, 5′-ACACAATTTGGCTCCATAGC-3′; *UBE2C* forward, 5′-GGATTTCTGCCTTCCCTGAA-3′; *UBE2C* reverse, 5′-GATAGCAGGGCGTGAGGAAC-3′; *GAPDH* forward, 5′-ACGGATTTGGTCGTATTGGGCG-3′; *GAPDH* reverse, 5′-CTCCTGGAAGATGGTGATGG-3′; *P53* forward: 5′-TCTTCCTCTGAGGCGAGCT-3′, *P53* reverse: 5′-AGGTGTGTGTGTCTGAGCCC-3′, *14-3-3A* forward: 5′-: GGCCATGGACATCAGCAAGAA-3′, *14-3-3A* reverse: 5′-CGAAAGTGGTCTTGGCCAGAG-3′, *NOXA* forward: 5′-AGAGCTGGAAGTCGAGTGT-3′, *NOXA* reverse: 5′-GCACCTTCACATTCCTCTC-3′.

### 4.5. Protein Extraction and Immunoblotting

To extract the total protein, cells were lysed using a Pro-Prep reagent (iNtRON Biotechnology, Seongnam, Korea). Each sample contained the same amounts of protein, as determined by quantifying the protein contents in the lysates. The samples were loaded and separated via SDS-polyacrylamide gel electrophoresis (PAGE). Proteins were transferred onto polyvinylidene difluoride (PVDF, Millipore, Temecula, CA, USA) membranes using the wet transfer method. The membranes were blocked using 5% skim milk for 1 h; then, they were incubated overnight with primary antibodies at 4 °C. After incubation with the primary antibodies, the membranes were incubated with horseradish peroxidase-conjugated secondary antibodies for 1 h (Millipore, Temecula, CA, USA). The signals were detected using chemiluminescence reagents (AbClon, Seoul, Korea) and quantified using ImageJ software.

### 4.6. Chromatin Immnoprecipitation (ChIP)-qPCR

Cells were crosslinked with 1% formaldehyde in PBS. Then, the crosslinked samples were sheared by sonification to obtain chromatin fragments that were 200–500 bp in size. The sheared chromatin fragments were incubated overnight at 4 °C with antibodies and magnetic beads, except for 2% input DNA. After immunoprecipitation, the chromatins were de-crosslinked at 65 °C, followed by the addition of RNase A and Proteinase K. DNA was purified from the samples and subjected to PCR as a template. The result was expressed as IP/Input (2%). The primers for the Chromatin Immunoprecipitation (ChIP) assay were the targets of the promoters of each gene, and the sequences were as follows: *CCNB1* forward, 5′-CGCGATCGCCCTGGAAACGCA-3′; *CCNB1* reverse, 5′-CGCGATCGCCCTGGAAACGCA-3′; *UBE2C* forward, 5′-CATTGGCTGGATCAAACCCA-3′; *UBE2C* reverse, 5′-GGAGAACACGACTGCAACTG-3′.

### 4.7. Wound Healing Assay

Cells were grown on 6 well plates until 100% confluence, followed by scratch with a 200 µL pipette tip. After washing with PBS to remove the floating cells, the cells were incubated in a medium with RAME, at concentrations of 0, 20 and 40 µM for 24 h. Gap width between the migrated cells was estimated in 4 random fields, under microscope, and averaged.

### 4.8. Transwell Migration Assay

A cell migration assay was performed using the Transwell chamber inserts (24-well; Corning Incorporated, Corning, NY, USA) in a 24-well plate. The upper membranes were coated with Geltrex (Life Technologies, Carlsbad, CA, USA) as a barrier (100 µL/well) for the invasion assay. Then, 5 × 10^4^ cells suspended in 100 µL of culture medium containing 1% FBS were added to the upper chamber. Culture medium containing 20% FBS was placed in the bottom chamber. The cells were incubated for 24 h at 37 °C. After incubation, the inserts were removed from the plate and fixed with 100% methanol for 2 min, followed by staining with crystal violet for 25 min. The cells on the upper surface of the insert were wiped off with a cotton swab. The cells that migrated to the lower layer of the microporous membrane were counted under a microscope. Five fields in each sample were randomly selected and the mean values were calculated.

### 4.9. Knockdown of FOXM1

SKOV-3 cells were transfected with siRNA using Lipofectamine 2000 reagent (Life Technologies, Carlsbad, CA), according to the manufacturer’s protocol. The siRNA sequences targeting FOXM1 were as follows:

FOXM1 sense: 5′-CCCUGCCCAACAGGAGUCUAAUCAA-3′,

FOXM1 antisense: 5′-UUGAUUAGACUCCUGUUGGGCAGGG-3′

### 4.10. Cell Viability Assay

A cell viability assay was performed using PrestoBlue™ (Invitrogen, Carlsbad, CA, USA; A13261) in a 96-well plate. The cancer cell lines were cultured at a density of 1 × 10^4^ cells per well. After incubation for 24 h at 37 °C, the cells were treated with 0–100 µM of cisplatin for 24 h, and the wavelength absorbance test was performed using Cytation™ 5 Cell Imaging Multi-Mode Reader (Biotek, Winooski, VT, USA).

### 4.11. Statistical Analysis

Statistical significance was analyzed using Student’s *t*-test (two-tailed) and Pearson’s correlation test. Statistical differences were assessed based on the following criteria for *P*-values: * *p* < 0.05, ** *p* < 0.01, and *** *p* < 0.001.

## 5. Conclusions

In conclusion, our study showed that RAME suppressed the expression of a novel molecular target, FOXM1, leading to anti-cancerous effects. By inhibiting the expression of the transcription factor FOXM1, the expression of its target genes was also downregulated. In fact, many transcription factors, such as Twist, CREB, and CTCF, are known to regulate the expression of FOXM1 by binding to its promoter [55]. Moreover, FOXM1 can be served as a transcription factor and can auto-regulate the expression of FOXM1 [56,57]. Therefore, further studies are needed to identify how RAME treatment regulates these various transcription factors, hence resulting in FOXM1 suppression. Our results also showed an effective decrease in the migration and invasion abilities of the ovarian cancer cells. Moreover, FOXM1 upregulation in cisplatin-resistant ovarian cancer cells was inhibited by RAME treatment. Consistently, co-treatment with RAME and cisplatin resulted in an increased sensitivity to chemotherapy and enhanced the apoptosis of cancer cells. These results showed that RAME could be an effective therapeutic agent to treat ovarian cancer patients, who also later develop chemoresistance. Additionally, further studies should be performed to elucidate the mechanism underlying the RAME-induced suppression of FOXM1 expression not only in ovarian cancer cells, but also in other cancer cells.

## Supplementary Materials

The following are available online at https://www.mdpi.com/1424-8247/13/10/302/s1. The RNA sequencing data are available from the Gene Expression Omnibus (GEO; GSE158604). Figure S1: FOXM1 expression level in ovarian cancer patients, Figure S2: Apoptotis marker gene expression by RAME treatment in ovarian cancer cell, Figure S3: The mRNA level of FOXM1 in SKOV-3 and TOV-21G cells; Table S1: The list of 242 down-regulated genes in RAME treated SKOV-3 ovarian cancer cells (|log2(Fold change)| > 1, *p*-value < 0.05).
