# Supplementary material for: Rosmarinic Acid Methyl Ester Regulates Ovarian Cancer Cell Migration and Reverses Cisplatin Resistance by Inhibiting the Expression of Forkhead Box M1
Source: Pharmaceuticals (Basel). 2020 Oct 12;13(10):302. doi: 10.3390/ph13100302 (PMC7601071; doi:10.3390/ph13100302)
Supplement: Supplementary file 1 [file pharmaceuticals-13-00302-s001.pdf]

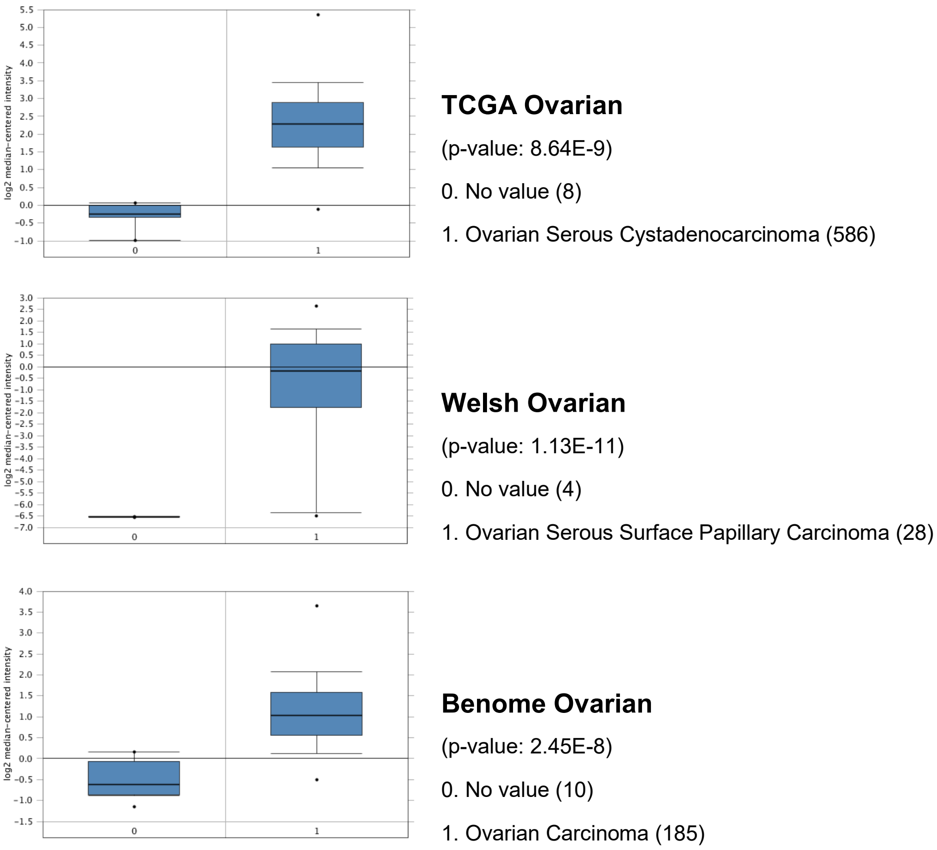

Figure 1. FOXM1 expression level in ovarian cancer patients. The relative mRNA expression of FOXM1 in TCGA cohort (Normal : 8, Cancer: 586) and two Oncomine dataset: Welsh Ovarian (Normal : 4, Cancer: 28) and Benome Ovarian (Normal : 10, Cancer: 185).

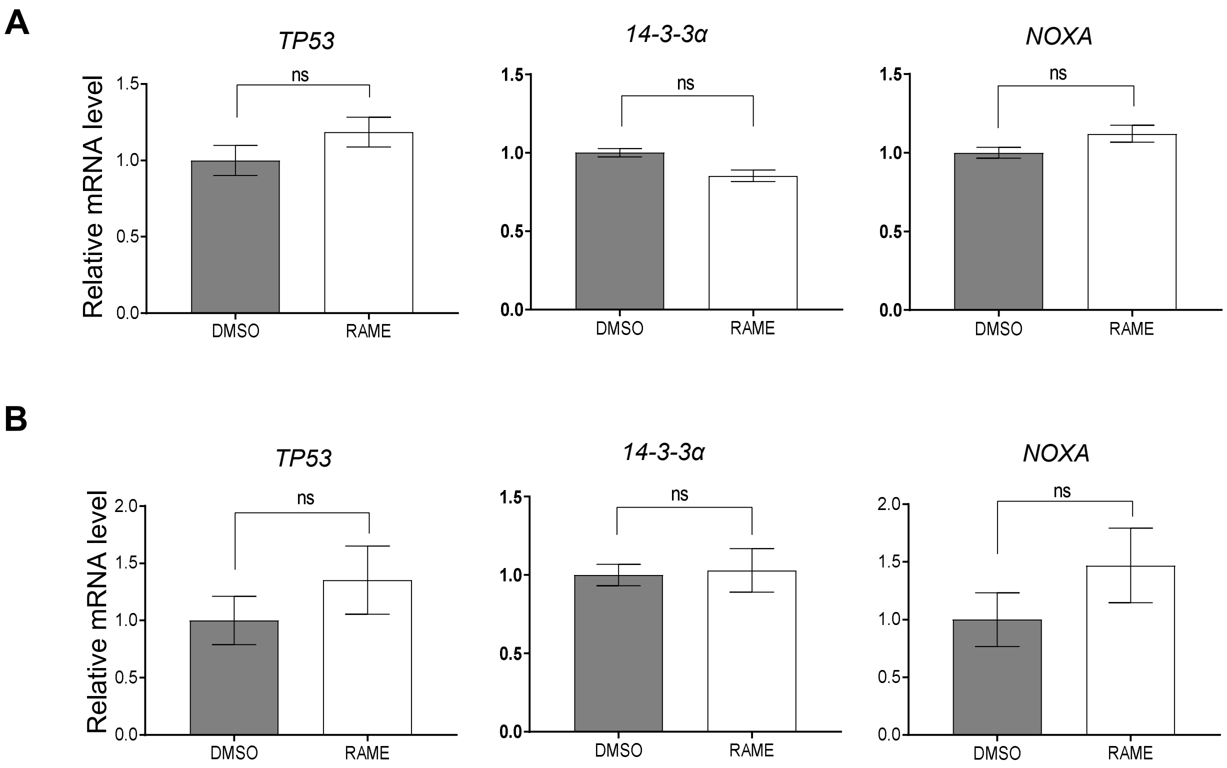

Figure 2. Apoptotis marker gene expression by RAME treatment in ovarian cancer cell. (A) The relative mRNA expression of apoptosis marker genes in SKOV-3 cells by RAME treatment (40μM). (B) The relative mRNA expression of apoptosis marker genes in TOV-21G cells by RAME treatment (40μM). Error bars represent the mean ± SEM (n = 3). *ns* : not significant.

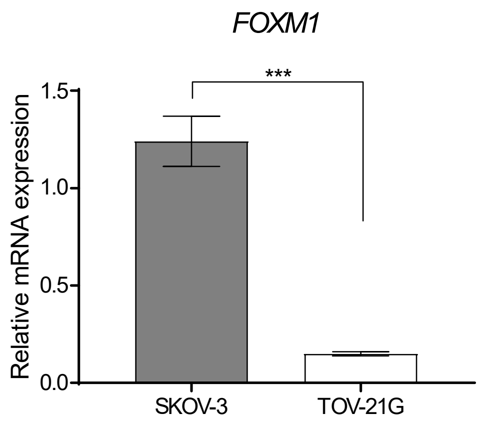

Figure 3. The mRNA level of FOXM1 in SKOV-3 and TOV-21G cells. The relative mRNA expression of FOXM1 in SKOV-3 and TOV-21G ovarian cancer cell lines. Error bars represent the mean ± SEM (n = 3). *p < 0.05, **p < 0.01, ***p < 0.001; unpaired t test.

Table 1. The list of 242 down-regulated genes in RAME treated SKOV-3 ovarian cancer cells (|log2 (Fold change)| > 1, *p*-value < 0.05).

	RAME / Con	
Gene symbol	Fold change	p-value
AMOTL2	0.063	0.000
CRTC2	0.086	0.027
TOP2A	0.098	0.033
SMARCAD1	0.107	0.021
PIF1	0.110	0.003
FOXO6	0.118	0.017
PPM1H	0.133	0.001
CCDC178	0.136	0.014
ELMSAN1	0.149	0.034
RUNX2	0.157	0.020
ZNF704	0.163	0.015
RBM12B	0.163	0.015
ATP5C1	0.185	0.013
LATS2	0.187	0.011
ADAM9	0.193	0.046
APITD1-CORT	0.196	0.016
POU3F1	0.196	0.016
ARMT1	0.197	0.005
POLR1A	0.198	0.039
CCNB1	0.203	0.020
ESR1	0.206	0.038
PLS1	0.207	0.002
KAT8	0.210	0.021
SPTBN4	0.210	0.018
SWT1	0.211	0.001
PRC1-AS1	0.212	0.033
TMEM168	0.221	0.005
NDUFAF4P1	0.229	0.008
TMEM203	0.229	0.008
FILIP1L	0.230	0.016
TMPO	0.232	0.013
NSMAF	0.244	0.007
NDST3	0.248	0.047
DHDDS	0.249	0.001
MAML2	0.256	0.009
GJA9-MYCBP	0.256	0.044
SFI1	0.257	0.010
PISD	0.257	0.010
ACVR2A	0.258	0.011
LARP1	0.261	0.021
ARID1A	0.264	0.004
ZNF518A	0.265	0.033
ZNRF3	0.265	0.012
GADD45GIP1	0.269	0.030
SORBS2	0.270	0.026
NAB1	0.270	0.008
ETS1	0.270	0.030
SKA3	0.271	0.041
FADS3	0.273	0.032
RGCC	0.273	0.032
UBE2C	0.273	0.032
HERC3	0.273	0.032
CENPH	0.273	0.032
WDR55	0.273	0.032
TTPAL	0.275	0.034
C11orf63	0.275	0.015
TMEM163	0.275	0.015
TTC17	0.277	0.002
CENPF	0.277	0.021
AKIRIN1	0.281	0.038
ZNF746	0.282	0.045
NAP1L3	0.282	0.040
STARD13	0.283	0.024
HOXA-AS3	0.285	0.003

EXTL3	0.289	0.032
H1FX	0.289	0.048
MARCKS	0.292	0.008
MKRN2	0.293	0.028
TWSG1	0.293	0.015
MYCBP	0.295	0.011
C16orf52	0.295	0.031
RAB10	0.295	0.002
NDUFAF5	0.295	0.035
CALML4	0.299	0.003
CDH7	0.300	0.027
CCDC112	0.301	0.037
GUCD1	0.304	0.003
HOXA5	0.304	0.001
TGFB3	0.305	0.001
SRBD1	0.305	0.001
WRN	0.305	0.001
CAV1	0.305	0.013
SIX4	0.309	0.040
BCL2	0.310	0.026
USP54	0.311	0.044
CTGF	0.312	0.041
CHIC2	0.315	0.010
MECOM	0.315	0.037
EXT1	0.315	0.025
RFX3	0.319	0.014
PDLIM5	0.320	0.017
ACSL1	0.321	0.039
VAPA	0.322	0.027
MPLKIP	0.322	0.010
WDR20	0.324	0.028
FAM129A	0.324	0.023
PPTC7	0.326	0.033
RAB28	0.331	0.005
ZZEF1	0.333	0.004
CDC73	0.335	0.010
TENM3	0.336	0.050
ACAT1	0.339	0.023
UBQLN2	0.340	0.014
KCTD20	0.341	0.037
RPRD2	0.341	0.049
SPRED1	0.343	0.017
KCTD1	0.344	0.022
MASP2	0.344	0.034
S1PR1	0.344	0.034
FAM72B	0.344	0.034
FHOD1	0.344	0.034
TSSK2	0.344	0.034
SENP7	0.344	0.034
MRS2	0.344	0.034
RAPGEF5	0.344	0.034
PTAR1	0.344	0.034
PAK1	0.348	0.046
FAM131A	0.352	0.038
ZNF319	0.354	0.010
IQGAP2	0.354	0.048
PKNOX1	0.355	0.007
CHTOP	0.355	0.034
SYNE1	0.356	0.037
RALGAPB	0.358	0.049
PNRC2	0.358	0.038
RDH10	0.362	0.039
RAF1	0.363	0.032
SP1	0.366	0.008
STK24	0.367	0.025
WDR5B	0.367	0.011
RFK	0.369	0.009
CCBE1	0.371	0.029
MECP2	0.373	0.038

RYR3	0.373	0.046
RP9P	0.375	0.039
ELK1	0.377	0.010
UBE2W	0.377	0.045
ZNF593	0.384	0.039
HTT	0.385	0.030
PIAS4	0.388	0.004
FGF13	0.389	0.028
NCKIPSD	0.391	0.014
LZIC	0.394	0.001
SLC25A24	0.394	0.001
MYRF	0.394	0.001
FOXM1	0.394	0.001
MRPS35	0.394	0.001
ZNF891	0.394	0.001
C14orf2	0.394	0.001
CRISPLD2	0.394	0.001
IRF3	0.394	0.001
ZNF444	0.394	0.001
CKAP2L	0.394	0.001
TMEM177	0.394	0.001
SLC35E4	0.394	0.001
LYRM7	0.394	0.001
PAK1IP1	0.394	0.001
AKAP7	0.394	0.001
TTC39B	0.394	0.001
FBXW5	0.394	0.001
ERCC6L	0.394	0.001
TCEAL4	0.394	0.001
RHOBTB3	0.394	0.034
MEX3A	0.396	0.008
AP3D1	0.398	0.018
ANKRD1	0.398	0.004
ZNF207	0.399	0.007
GNPDA1	0.399	0.043
NRIP1	0.406	0.010
ZNRF1	0.407	0.010
TCF20	0.407	0.015
KLF3	0.407	0.038
SNRNP70	0.408	0.045
LUC7L3	0.411	0.027
ARIH1	0.413	0.006
E2F5	0.413	0.007
C8orf59	0.417	0.009
TMEM87A	0.417	0.012
CDC27	0.418	0.014
OCRL	0.419	0.018
TNFRSF12A	0.421	0.015
SNIP1	0.422	0.000
VCL	0.422	0.019
QKI	0.423	0.005
ZADH2	0.425	0.045
CNIH1	0.429	0.029
BRPF3	0.430	0.004
GLIS2	0.430	0.035
PRPF4B	0.430	0.015
LRP1	0.431	0.039
CCZ1B	0.432	0.014
ANKH	0.433	0.014
CCNY	0.435	0.037
NAA30	0.435	0.022
CRKL	0.436	0.039
FOXP1	0.439	0.028
RND1	0.440	0.007
KCTD5	0.440	0.037
MED13L	0.441	0.021
DDX49	0.441	0.011
TNPO1	0.444	0.016
ARID5B	0.447	0.037

CKAP5	0.450	0.036
STRN3	0.451	0.044
CDC42SE2	0.451	0.015
DDX52	0.452	0.036
PPIL4	0.453	0.012
BMPR2	0.453	0.012
GRB2	0.455	0.046
ING5	0.456	0.045
DMD	0.456	0.025
PIGW	0.460	0.005
NFIB	0.461	0.009
PUM2	0.462	0.010
NUP153	0.462	0.026
CEPT1	0.464	0.007
PPRC1	0.470	0.049
IMMP1L	0.470	0.025
TRPC1	0.471	0.032
DNAJC24	0.473	0.024
CUL5	0.473	0.014
GLS	0.474	0.017
B3GNT9	0.475	0.001
TRIM8	0.478	0.022
G3BP1	0.480	0.004
ILF3	0.480	0.006
FAM179B	0.481	0.006
VEZF1	0.482	0.021
MBNL1	0.484	0.013
TRPS1	0.486	0.013
CAMK2D	0.487	0.022
SYNCRIP	0.487	0.018
SIRT1	0.487	0.025
CHFR	0.493	0.008
BAZ2B	0.494	0.036
GSK3B	0.495	0.022
YY1	0.495	0.009
NR2F2	0.495	0.029
SP2	0.498	0.003
MIER3	0.498	0.018
LMO4	0.499	0.016
PEG3	0.500	0.048
